# Association between diabetes mellitus and depressive symptoms in the Brazilian population

**DOI:** 10.11606/S1518-8787.2019053000608

**Published:** 2018-12-20

**Authors:** Cauê Pontes Briganti, Marcus Tolentino Silva, José Vanilton de Almeida, Cristiane de Cássia Bergamaschi

**Affiliations:** I Universidade de Sorocaba. Programa de Pós-Graduação em Ciências Farmacêuticas. Sorocaba, SP, Brasil; II Universidade Federal do Amazonas. Faculdade de Medicina. Manaus, AM, Brasil

**Keywords:** Adult, Diabetes Mellitus, epidemiology, Depression, epidemiology, Risk factors, Patient Health Questionnaire, utilization, Health Surveys

## Abstract

**OBJECTIVE:**

To determine the prevalence of current depressive symptoms in people with diabetes mellitus and their association with the disease.

**METHODS:**

Data were collected from the Brazilian National Health Survey (Pesquisa Nacional de Saúde - PNS), a cross-sectional, population-based study conducted in 2013. Study participants were selected by simple random cluster sampling in three stages: census tracts, households, and residents aged ≥ 18 years. The presence of diabetes was self-reported, whereas the presence of current depressive symptoms was determined by the Patient Health Questionnaire-9 (PHQ-9) and mean scores of this questionnaire were calculated for the variables assessed. Tobit regression was used to evaluate variation in these individuals.

**RESULTS:**

Of the 60,202 interviewees, 6.03% (n = 3,636) reported diabetes mellitus. The disease was more frequent in female, older, widowed, obese and with incomplete elementary education. Depression symptoms were mild-to-moderately severe in 22% of the diabetics. The severity of current depressive symptoms was higher in individuals that were female (PHQ-9 mean = 3.35), older adults (PHQ-9 mean = 3.01), indigenous (PHQ-9 mean = 3.46), separated/divorced (PHQ-9 mean = 3.13), widowed (PHQ-9 mean = 3.39), obese (PHQ-9 mean = 3.13) and with incomplete primary education (PHQ-9 mean = 3.21). Higher severity of depressive symptoms was associated with the use of insulin and with coma (PHQ-9 mean = 8.32), limb amputation (PHQ-9 mean = 7.55), circulatory problems (PHQ-9 mean = 6.94), infarction (PHQ-9 mean = 6.83), diabetic foot (PHQ-9 mean = 6.62), and kidney problems (PHQ-9 mean = 6.68). The severity of current depressive symptoms was associated with diabetes severity and degree of limitation in activities of daily living (PHQ-9 mean = 10.62).

**CONCLUSIONS:**

Interventions to improve depressive symptoms should be prioritized in people with diabetes are female, older adults, indigenous, widowed, separated/divorced, obese and with incomplete elementary education.

## INTRODUCTION

Diabetes is a chronic, progressive disease often associated with numerous neuropsychiatric comorbidities, particularly depression. The prevalence of depression is 2-3 times higher in patients with diabetes than in the general population^1^ ^,^ ^2^. Depression is considered one of the most overlooked symptoms in diabetics and is directly associated with a worse quality of life^2^.

According to the International Diabetes Federation, there were 382 million people with type 2 diabetes mellitus in 2013^3^. Epidemiological studies in the United Nations’ countries found a global prevalence of diabetes of 8.3% among adults aged 20-79 years in 2011, with an estimated increase to 9.9% by 2030^4^.

A systematic review showed that depression rates among people with diabetes in low- and middle-income countries may be higher than that in high-income countries^3^. Another systematic review of data from high-income countries indicated that depression contributes to increased diabetes-related comorbidities and higher health system costs^5^. Yet another showed that people with type 2 diabetes have a 24% higher likelihood of developing depressive symptoms in comparison to individuals without the disease^6^.

The severity of depressive episodes, dysthymia, mood disorders, and suicidal ideation is associated with lower quality of life, increased comorbidities, and poor diabetes control^7^. There are several obstacles to detecting cases using population-based surveys, including the lack of a gold standard and the high level of resources required to conduct two-step interviews. Consequently, validated instruments measuring depressive symptoms at the time of interview tend to be employed^8^.

A cross-sectional study evaluated the prevalence of depressive and anxiety symptoms together with the decline in quality of life in 210 individuals with type 1 and 2 diabetes. The authors found a higher prevalence of anxiety and depression in patients with type 1 diabetes. In general, individuals with symptoms of depression and anxiety had worse quality of life^9^.

A Brazilian hospital survey of 346 individuals with type 1 and 2 diabetes demonstrated that patients with type 1 diabetes had a higher quality of life score than patients with type 2 diabetes regarding mobility, self-care, performance of activities of daily living, pain and discomfort, and anxiety and depression^10^.

In Brazil, diabetes prevalence is generally estimated by health surveys based on self-reported measures owing to difficulties collecting biochemical data in large populations^11^. The National Health Survey (Pesquisa Nacional da Saúde - PNS) was carried out by the Brazilian Ministry of Health and described the health situation and lifestyles of Brazilians, as well as their healthcare in terms of access and use of services, preventive actions, continuity of care, and healthcare expenditure^12^. This population-based survey determined the prevalence of self-reported diabetes in the Brazilian population.

Data from the National Health Survey in 2013 showed that 4.1% of the Brazilian population currently had depressive symptoms. The authors observed that depression was more frequent among individuals that were female, aged ≥ 40-59 years, living in urban areas, less educated, smokers, hypertensive, diabetic and with heart disease^13^.

The mental health state of the population is an important and essential component of public health since depression is the most prevalent mental disorder worldwide^13^. In Brazil, there are no epidemiological studies determining the prevalence of depression among diabetic individuals or the factors associated with the condition. This cross-sectional study sought to determine the prevalence of current depressive symptoms in diabetes mellitus and their association with the disease.

## METHODS

### Study Design

This is a cross-sectional study drawing on the National Health Survey, a population-based household survey with national representativeness, conducted in 2013. Self-reported diabetes mellitus and current depressive symptoms were the primary outcomes analyzed.

### Research Site and Setting

The National Health Survey was carried out by the Brazilian Institute of Geography and Statistics, in collaboration with the Ministry of Health, and entailed interviews conducted with adults, collecting data on their health status, access to public services, participation in prevention activities, and lifestyles^12^.

Households were drawn from the master sample of the National Household Sample Survey, an annual survey used by the IBGE for planning Federal Government actions. This approach provided greater geographic coverage and improved accuracy of estimates^14^.

Methodological details, including the survey design and development process, have been described by Damacena et al.^12^ and Souza-Júnior et al.^15^

### Sample Selection

The National Health Survey adopted simple randomized cluster sampling in three stages: census tracts, households, and residents aged ≥ 18 years^14^. The total sample size was 81,357 households. In the first stage of the random selection process, sectors with special characteristics and low population (indigenous, barracks, military bases, lodgings, camps, boats, penitentiaries, penal colonies, prisons, asylums, orphanages, convents, and hospitals) were excluded^16^. Thus, 81,167 households were visited, of which 69,994 were occupied. A total of 60,202 individual interviews were conducted with the randomly selected residents^13^. For this study, interviews containing answers to questions about the presence of diabetes mellitus and depressive symptoms were selected.

### Data Collection Procedure

The individual questionnaires were completed with the aid of handheld computers by interviewers previously trained by the IBGE and Ministry of Health. The results are available for download on the IBGE website (http://www.ibge.gov.br/home/estatistica/populacao/pns/2013_vol3/default_microdados.shtm).

Diabetes prevalence was self-reported and addressed by the question: “Has a doctor ever diagnosed you with diabetes?”. The presence and severity of current depressive symptoms in the population with diabetes were measured by the Patient Health Questionnaire-9 (PHQ-9)^17^. Such questionnaire comprises nine questions scored as zero (no days), one (less than half the days), two (more than half the days) and three (almost every day). Interviewees were stratified by the severity of current depressive symptoms according to the PHQ-9 score: none-minimal (0-4 points), mild (5-9 points), moderate (10-14 points), moderately-severe (15-19 points), and severe (20-27 points).

To characterize the sample, the following information was extracted from the database: gender (male, female), age (18-39, 40-59, ≥ 60), ethnicity (white, black, Asian, brown, or indigenous), marital status (married, separated or divorced, widowed, and single), education (higher education or post-secondary education, secondary school, elementary school, incomplete elementary school), Body Mass Index - BMI (normal weight, overweight, obese), strategies used in diabetes treatment, habitual activities, and the severity of current depressive symptoms in the population.

Regarding treatment strategies, participants were asked whether they regularly visited the doctor for diabetes mellitus, received treatment guidelines, were routinely examined for diabetes-related complications (visual problems, infarction, stroke, other circulatory problems, kidney problems, diabetic foot, upper and lower limb amputation, diabetic coma, and others) and whether they had been hospitalized for diabetes. Limitations in habitual activities due to diabetes were measured by a Likert-type scale (not limited, a little, moderately, severely, and very severely).

### Statistical Analysis

Presence of diabetes and the PHQ-9 score were used for quantitative analyses. The participants’ characteristics were described using simple frequencies and stratified by the presence of diabetes and severity of current depressive symptoms. Treatment strategies frequency, the degree of limitation in habitual activities, and severity of current depressive symptoms were described for the diabetic population.

PHQ-9 mean values were calculated for the variables studied. Considering the ceiling effect of the PHQ-9 score (most people do not present depressive symptoms and have zero scores), Tobit regression was used to evaluate score variation in patients with diabetes. The effect of complex sampling was considered for all calculations and associations were regarded as significant when p < 0.05. Analyses were performed using the statistical program Stata (version 14.2). Sensitivity analysis was performed using a bootstrap technique.

### Ethics Approval and Participant Consent

The National Health Survey was approved by the National Ethics Commission on July 8, 2013, under Protocol 10853812.7.0000.0008. Adult participation in the survey was voluntary and the confidentiality of information was ensured, in accordance with Resolution no. 466 of December 12, 2012, of the National Health Council.

## RESULTS

In a population of 60,202 respondents, 3,636 (6.03%) had diabetes. The total population consisted predominantly of females, individuals aged 18-39 years, white and brown, married, single or widowed, educated until the high school level, and non-obese.

The population with diabetes mellitus comprised predominantly individuals that were female, older adults, widowers, with incomplete primary education and obese. Moderately-severe or severe current depressive symptoms were also higher in this group, although most had none or minimal depressive symptoms. Approximately 22.5% of participants had mild- to-severe current depressive symptoms (Table 1).

**Table 1:** Sociodemographic and clinical characteristics of the Brazilian population with *diabetes mellitus* as a function of depressive symptoms. Brazil, 2013. (n = 3,636)

Variable		Population				Depressive symptoms				
		Total n (%)	Diabetes n (%)	p	% None-minimal	% Mild	% Moderate	% Moderately severe	% Severe	p
Gender										
	Male	25,916 (43.0)	1,281 (6.3)	0.0025	85.43	9.85	3.00	1.14	0.58	< 0.0001
	Female	34,286 (56.9)	2,355 (7.6)		73.08	16.20	6.37	2.90	1.45	
Age (years)										
	18-39	28,722 (47.7)	315 (1.2)	< 0.001	81.25	12.32	4.07	1.63	0.73	< 0.0001
	40-59	20,613 (34.2)	1,425 (7.9)		76.73	14.09	5.50	2.32	1.36	
	≥ 60	10,866 (18.0)	1,896 (19.1)		76.81	13.86	5.33	2.76	1.25	
Ethnicity*										
	White	28,571 (47.5)	1,556 (7.3)	0.0779	79.53	12.98	4.47	2.08	0.94	< 0.1241
	Black	5,538 (9.2)	415 (8.2)		79.08	12.36	5.21	2.02	1.33	
	Asian	565 (0.94)	29 (6.8)		79.39	12.93	4.38	2.29	1.00	
	Brown	25,272 (41.9)	1,608 (6.5)		78.22	13.56	5.06	2.05	1.11	
	Indigenous	252 (0.4)	27 (8.5)		69.66	22.24	5.16	2.82	0.13	
Marital status										
	Married	26,669 (44.3)	1,621 (8.3)	< 0.001	79.84	12.32	4.71	2.08	1.05	< 0.0015
	Separated/Divorced	3,913 (6.5)	416 (9.8)		75.30	14.67	6.09	2.43	1.51	
	Widowed	4,021 (6.7)	757 (17.5)		73.40	15.78	6.42	3.17	1.23	
	Single	25,597 (42.5)	842 (3.2)		79.33	13.50	4.40	1.83	0.94	
Education*										
	Higher or postgraduate	9,228 (15.3)	365 (4.3)	< 0.001	81.35	12.95	3.75	1.44	0.50	< 0.0001
	High School	23,575 (39.2)	730 (3.8)		81.55	12.24	3.99	1.53	0.69	
	Elementary School	2,516 (4.2)	1,788 (9.7)		77.09	13.61	5.42	2.35	1.52	
	Incomplete elementary school	2,233 (3.7)	216 (11.5)		75.13	14.90	5.43	3.29	1.25	
Body Mass Index*										
	Normal weight (18.5-25 kg/m^2^)	27,801 (46.2)	602 (3.8)	< 0.001	80.72	12.52	4.37	1.50	0.89	< 0.0001
	Overweight (≥ 25 kg/m^2^)	21,161 (35.1)	931 (7.5)		80.81	12.64	4.11	1.65	0.78	
	Obese (≥ 30 kg/m^2^)	1 1,239 (1 8.7)	823 (12.4)		74.98	15.02	5.94	3.06	1.00	

* Not all participants responded.

The presence of current depressive symptoms in the population with diabetes mellitus was higher in women, and especially among older adults, indigenous, separated/divorced and widowed, obese individuals and those with incomplete primary education (Table 2).

**Table 2:** Variables predicting depressive symptoms in the Brazilian population with diabetes mellitus. Brazil, 2013. (n = 3,636)

Variable		Mean PHQ-9	Coefficient	p
Gender				
	Male	1.97	Ref	
	Female	3.35	2.59	< 0.001
Age (years)				
	18-39	2.40	Ref	
	40-59	2.96	0.99	< 0.001
	≥ 60	3.01	1.15	< 0.001
Ethnicity				
	White	2.62	Ref	
	Black	2.74	0.19	0.351
	Asian	2.64	-0.05	0.927
	Brown	2.78	0.36	0.020
	Indigenous	3.46	1.55	0.011
Marital status				
	Married	2.63	Ref	
	Separated/Divorced	3.13	0.91	< 0.001
	Widowed	3.39	1.41	< 0.001
	Single	2.60	0.00	0.991
Education				
	Higher or postgraduate	2.32	Ref	
	High School	2.34	0.05	0.739
	Elementary School	2.99	1.17	< 0.001
	Incomplete elementary school	3.21	1.66	< 0.001
Body Mass Index				
	Normal weight (18.5-24.99 kg/m^2^)	2.49	Ref	
	Overweight (≥ 25 kg/m^2^)	2.44	-0.19	0.171
	Obese (≥ 30 kg/m^2^)	3.13	1.00	< 0.001

Ref: reference; PHQ-9: Patient Health Questionnaire-9

The severity of current depressive symptoms was evaluated in relation to treatment strategies, duration of illness, and diabetes complications. Patients with diabetes diagnosed within the last two years had the greatest severity of depressive symptoms. The frequency of medical visits for diabetes was not associated with severity of depressive symptoms. The use of insulin was associated with higher severity of depressive symptoms compared with the use of oral medications. Mean values of the PHQ-9 were higher in individuals with coma, limb amputation, circulatory problems, infarction, diabetic foot, and kidney problems. The severity of current depressive symptoms was higher the greater the limitation in habitual activities caused by diabetes (Table 3).

**Table 3:** Treatment strategies, diabetes complications, and limitation of habitual activities as a function of depressive symptoms severity in the population with diabetes mellitus. Brazil, 2013. (n = 3,636)

Variables		% Diabetes	% None-minimal	% Mild	% Moderate	% Moderately severe	% Severe	p	Mean PHQ-9	PHQ-9 Coefficient	p
Duration of illness (years)											
	0-2	27.8	66.7	18.1	8.9	4.4	1.9	0.8471	4.22	Ref	
	3-7	24.8	68.3	16.9	7.7	5.3	1.8		4.05	-0.28	0.659
	8-15	23.5	67.6	19.8	6.2	3.8	2.7		3.99	-0.37	0.562
	≥ 16	23.9	68.8	15.8	6.9	5.3	3.3		4.19	-0.37	0.603
Use of oral medication		75.2	68.1	17.2	7.2	5.1	2.4	0.6740	4.09	-0.19	0.712
Use of insulin		17.8	62.2	16.1	9.2	8.9	3.7	0.0082	5.25	2.03	0.002
Do you regularly visit the doctor for diabetes											
	Yes	67.7	68.1	16.98	7.28	5.18	2.46	0.3035	4.17	Ref	
	No (only if you have problem)	23.0	69.81	17.64	7.32	2.64	2.58		3.76	-0.73	0.223
	Never visit	9.3	60.68	22.39	9.28	6.41	1.25		4.61	0.57	0.458
Complications of diabetes											
	Visual problems	31.9	60.3	18.5	11.0	6.1	4.1	0.0004	5.26	2.41	< 0.001
	Infarction	4.3	48.4	21.2	7.3	20.5	2.6	< 0.0001	6.83	3.34	0.029
	Stroke	3.9	39.9	36.3	13.5	8.4	2.0	0.0002	6.14	3.31	< 0.001
	Other circulatory problems	13.7	49.6	20.4	13.2	8.8	8.1	< 0.0001	6.94	4.36	< 0.001
	Kidney problem	12.3	50.2	20.5	14.3	8.8	6.2	< 0.0001	6.68	3.82	< 0.001
	Diabetic foot	6.1	51.1	21.9	10.6	11.9	4.5	0.0484	6.62	3.37	0.008
	Amputation of upper or lower limbs	1.6	52.7	13.2	11.0	1.3	21.8	< 0.0001	7.55	3.55	0.215
	Diabetic coma	2.1	40.6	29.3	9.5	5.5	15.1	< 0.0001	8.32	5.51	0.001
	Other	1.8	60.7	19.8	11.1	3.9	4.5	0.7758	5.18	1.89	0.163
	Hospitalization	15.4	54.2	19.1	10.6	12.2	3.9	< 0.0001	6.19	3.34	< 0.001
Diabetes or complication of diabetes limit your habitual activities											
	Does not limit	66.5	75.2	15.3	5.8	2.6	1.2	< 0.0001	3.12	Ref	
	Limit a little	17.5	59.6	23.8	8.9	5.2	2.4		4.87	2.75	< 0.001
	Moderately	9.0	54.9	21.2	9.8	9.2	4.9		5.95	4.10	< 0.001
	Severely	5.1	37.1	21.3	13.1	21.6	6.9		8.80	7.15	< 0.001
	Very severely	2.0	28.1	17.2	27.6	8.2	18.9		10.62	9.34	< 0.001

Ref: Reference; PHQ-9: Patient Health Questionnaire-9

## DISCUSSION

### Summary of Findings and Interpretation Based on Available Literature

In this study, one in every 16 respondents was diagnosed with diabetes mellitus, corresponding to approximately 6% of the population. The diabetes group comprised predominantly women, older adults, divorced or separated individuals with incomplete elementary education and obese status. Approximately 22% of the patients with diabetes had current depressive symptoms, whose severity was higher in women, older adults, indigenous, separated/divorced and widowed individuals with incomplete primary education and obese status.

Diabetic patients diagnosed within the last two years and in use of insulin had greater severity of current depressive symptoms. In addition, the presence of comorbidities (coma, limb amputation, circulatory problems, infarction, diabetic foot and kidney problems) was associated with greater severity of depressive symptoms. When the disease severely limited the activities of individuals with diabetes, this was also associated with a greater severity of depressive symptoms.

A 2016 population-based study conducted in Spain, involving 320 interviews with type 2 diabetes mellitus, found that 27.2% had depressive symptoms (as measured by the Spanish version of the Beck Depression Inventory). These symptoms predominantly affected women, single individuals and the unemployed^18^; similar to the pattern observed in this study.

An observational study conducted between 2013 and 2014 in Pakistan identified the presence of depressive symptoms (according to PHQ-9 and the Diagnostic and Statistical Manual of Mental Disorders) in 133 (38.35%) patients with type 2 diabetes mellitus. Most patients were women, had low education, shorter time since diabetes diagnosis and obese status^19^. Although the population of the cited study was smaller than that of this investigation, the profile of patients was similar - female gender, low education, obesity status and a short time since diagnosis.

Another cross-sectional study evaluating the prevalence of depression in 80 patients with type 2 diabetes hospitalized in the south of India found that 38.75% of subjects with type 2 diabetes were diagnosed with depression (based on the Diagnostic and Statistical Manual of Mental Disorders). One should note that none of the factors evaluated (age, sex, education, income, and marital status) had a significant influence on the occurrence of depression; this, according to the author, may have been due to the small sample size, which precluded the generalization of findings^20^.

A cross-sectional study carried out in Australia involving 5,462 older men with self-reported diabetes showed that 6.5% had depressive symptoms (according to the Geriatric Depression Scale - GDS15). These older individuals were nearly twice as likely to have depression compared with those without diabetes (OR = 1.94, 95%CI 1.15-2.48)^21^. Although the study was specifically in a male population, it demonstrated the presence of depressive symptoms in older adults with diabetes, a finding also observed in the Brazilian population.

In a cross-sectional study with 700 participants, conducted at three public clinics in Malaysia in 2013, the prevalence of any degree of depression was 41.7%, with frequent mild depressive symptoms in 30.6% of patients. These symptoms were more prevalent in individuals who were single, divorced/separated and with microvascular complications (retinopathy, nephropathy, and diabetic foot)^22^. In this study, some of the complications of diabetes (coma, limb amputation, circulatory problems, infarction, and the presence of diabetic foot and kidney problems) were also associated with increased severity of depressive symptoms.

A cross-sectional multicenter study carried out at four outpatient clinics in Pakistan included 889 adults with type 2 diabetes. Approximately 43% of the participants had depression, and this condition was significantly associated with being female, older, having ischemic heart disease and obese status^23^. Such result was similar to the findings of this study, with depression being found predominantly in females, older adults, and obese participants.

Many studies, including systematic reviews, have found that people at risk of having diabetes and diabetics have more depressive symptoms compared with non-diabetics^24^ ^,^ ^25^, and the literature has shown depression is a risk factor for developing diabetes. A systematic review observed that depressed individuals have a 41% greater risk for developing diabetes mellitus and a 32% greater risk for developing type 2 diabetes. However, the authors noted that the mechanisms underlying this relationship remain unclear and warrant further research^26^.

A study conducted in Romania with 1,171 diabetic patients, using the PHQ-9 and the Diabetes Costs Questionnaire (DCQ), assessed direct and indirect costs related to diabetes. This study showed statistically significant higher diabetes-related costs for patients with severe or mild depression than for patients without depressive symptoms^27^.

It is important emphasize that published data, also based on the National Health Survey (2013), showed that 4.1% of the Brazilian population had current depressive symptoms^13^, a lower prevalence than the rate found in the Brazilian population with diabetes; confirming the findings of the literature and showing the negative impact of diabetes on the living of the Brazilian population.

### Validity of the Study: Limitations and Strengths

Due to the cross-sectional design of this study, home interviews have been biased by including only participants who were at home at the time of data collection. In addition, it should be considered that depressed patients might be less interviewed, leading to underestimation of the association found.

On the other hand, the sample size is a strength of this study. The National Health Survey is a nationwide, community-based epidemiological survey that includes Federal and capital units, metropolitan areas, and the rest of the Federal units in the country. This study was based on the master sample of such system, which provided wide geographical coverage and improved the accuracy of estimates^14^ ^,^ ^16^.

Some methodological limitations inherent to prevalence studies, and the fact that diabetes mellitus was self-reported, may have led to disease underdiagnoses. The data was therefore insufficient for allowing diabetes to be categorized. However, for a survey of this magnitude, it is impractical to collect data in two steps to confirm the diagnosis.

In addition, the use of a validated instrument to assess depressive symptoms (PHQ-9) demonstrates the care taken in devising the questionnaire. However, it should be pointed out that the measure of depressive symptoms was based on the 2-week period leading up to interview and, therefore, provides only a current prevalence of depression. Lastly, it is important to emphasize that the prevalence of depression reported by studies may vary owing to differences in assessment methods (self-reported, screening instruments, clinical diagnosis etc.), sample type (population-based, hospital in-patients etc.), among other factors.

### Implications for Clinical Practice and Research

Cross-sectional studies are important for collecting up-to-date information on the population with diabetes and can aid health service planning and future research. In this respect, this study is important given the absence of electronic databases containing this much-needed information.

The creation of strategies to improve depressive symptoms in individuals with diabetes mellitus should prioritize women, older individuals indigenous, widowed, separated/divorced, obese and with incomplete elementary education.

The information yielded by this study may contribute to a better understanding of depression and its associated factors in the Brazilian population with diabetes mellitus and help guide initiatives and programs promoting care in this group of patients.
